# Suppression of Interferon Response and Antiviral Strategies of Bunyaviruses

**DOI:** 10.3390/tropicalmed9090205

**Published:** 2024-09-07

**Authors:** Yingying He, Min Shen, Xiaohe Wang, Anqi Yin, Bingyan Liu, Jie Zhu, Zhenhua Zhang

**Affiliations:** 1 Institute of Clinical Virology, Department of Infectious Diseases, The Second Affiliated Hospital of Anhui Medical University, Hefei 230601, China; hyy0189@163.com (Y.H.); shenmin200202@163.com (M.S.); wxhwxh2001@outlook.com (X.W.); yaq0127@163.com (A.Y.); lby081921@163.com (B.L.); 2 Department of Clinical Medicine, Anhui Medical University, Hefei 230032, China

**Keywords:** bunyavirus, interferon, NSs, TBK1, STAT, targeted therapy

## Abstract

The order Bunyavirales belongs to the class of Ellioviricetes and is classified into fourteen families. Some species of the order Bunyavirales pose potential threats to human health. The continuously increasing research reveals that various viruses within this order achieve immune evasion in the host through suppressing interferon (IFN) response. As the types and nodes of the interferon response pathway are continually updated or enriched, the IFN suppression mechanisms and target points of different virus species within this order are also constantly enriched and exhibit variations. For instance, Puumala virus (PUUV) and Tula virus (TULV) can inhibit IFN response through their functional NSs inhibiting downstream factor IRF3 activity. Nevertheless, the IFN suppression mechanisms of Dabie bandavirus (DBV) and Guertu virus (GTV) are mostly mediated by viral inclusion bodies (IBs) or filamentous structures (FSs). Currently, there are no effective drugs against several viruses belonging to this order that pose significant threats to society and human health. While the discovery, development, and application of antiviral drugs constitute a lengthy process, our focus on key targets in the IFN response suppression process of the virus leads to potential antiviral strategies, which provide references for both basic research and practical applications.

## 1. Introduction

The order Bunyavirales belongs to the class of Ellioviricetes. Over 500 different species of bunyaviruses were described and are widely distributed around the world. They circulate in nature through blood-feeding arthropods and susceptible vertebrates with the capability to infect a variety of animals and plants [1], which leads to significant biosecurity concerns and causes severe economic damage. Among them, some important bunyaviruses, such as Crimean–Congo hemorrhagic fever virus (CCHFV) and Dabie bandavirus (DBV), are primarily transmitted through tick biting, while La Crosse virus (LACV) is mainly transmitted by the Aedes triseriatus mosquito, etc.

According to the International Committee on Taxonomy of Viruses (ICTV), the order Bunyaviridaes currently is divided into fourteen families: Arenaviridae, Cruliviridae, Discoviridae, Fimoviridae, Hantaviridae, Leishbuviridae, Mypoviridae, Nairoviridae, Peribunyaviridae, Phasmaviridae, Phenuiviridae, Tospoviridae, Tulasviridae, and Wupedeviridae. Some viruses belonging to the order Bunyaviridaes can infect humans and lead to various disease syndromes, including febrile illness, encephalitis, hemorrhagic fever, and acute respiratory disease [2]. The family Tospoviridae is made up of viruses that only infect plants. Some bunyaviruses have a large impact on human health. For example, Crimean–Congo hemorrhagic fever (CCHF) occurs sporadically in many regions of Africa, Asia, and Europe with a mortality rate of approximately 30% [3]. The main clinical manifestations of severe fever with thrombocytopenia syndrome (SFTS) caused by novel bunyavirus are high fever, thrombocytopenia, leukopenia, gastrointestinal symptoms, liver and kidney dysfunction, and even multiple organ dysfunction syndrome (MODS). Although LACV infection is rarely fatal (<1%), it may lead to long-term neurological sequelae such as seizures, hemiparesis, or cognitive/neurobehavioral abnormalities [4]. Due to the lack of specific antiviral treatments or vaccines, bunyavirus infections continue to pose a global threat to human health, affecting various populations in both developing and developed countries and potentially leading to outbreaks, which is a significant public health concern [5].

Interferon (IFN) is an important cytokine produced by the body when infected with viruses and can trigger antiviral immune responses. However, numerous studies show that a variety of bunyaviruses can inhibit IFN response, especially the type I IFN pathway [2]. Bunyaviruses evade the recognition and attack of the host by inhibiting IFN production and signal transduction to complete the initial replication and spread after successfully invading the host, which is closely related to the severity and death of the disease [6,7]. In recent years, researchers not only explored the mechanism of how bunyaviruses inhibit the signaling pathway of IFN production, but also revealed many key targets related to the regulation of IFN response that have potential prospects for antiviral drug development. In this paper, we review the current studies on the suppression of IFN response in bunyavirus infections. Furthermore, we summarize and predict the potential therapeutic approaches aiming at the key targets of IFN response that may be used for bunyavirus infections by countering the viral suppression of IFN responses.

## 2. Antiviral Effects of the IFN Response

IFN belongs to a family of cytokines that was first described in 1957 as the inhibitor of viral replication [8]. They not only play a role in various cancers and infections but also are capable of immunomodulation and have a major impact on the development and maintenance of innate and adaptive immunity [9,10,11,12]. IFN plays an essential role in the fight against various infections, especially as an anti-virus [13,14,15,16,17,18]. As a broad-spectrum antiviral agent, it inhibits both RNA and DNA viruses by inducing the expression of IFN-stimulated genes (ISGs). It mediates antiviral effects through viral RNA degradation, viral translation inhibition, or both. IFN can also directly activate immune cells and indirectly inhibit the replication process of viruses during early viral infections in the organism.

Based on the amino acid sequences of IFNs, they are categorized into three types, type I (IFNα, IFNβ, IFNδ, IFNε, IFNκ, IFNτ, IFNω, and IFNζ), type II (IFNγ), and type III (IFNλ) [19]. Among them, type I and type III IFNs are classic antiviral IFNs with highly similar reaction pathways. Type I IFNs have a protective effect in virus systemic infection, whereas the antiviral effect of type III IFNs is particularly pronounced at epithelial barriers such as the gastrointestinal, respiratory, and reproductive tracts [20,21]. Moreover, type I IFN response is rapid and strongly induced ISG production, while type III IFN induces a relatively slow and stable ISG response [22]. Unlike other IFNs, the main function of type II IFN is immunomodulatory and its antiviral effect is weak. However, it plays a special role in the long-term control of viral infection [13].

Type I IFNs have the largest number of family members, and the main subtypes studied are IFNα and IFNβ, which are also the most reported to play a role in antiviral responses. Type I IFNs are synthesized very rapidly because of the absence of introns in their gene structure and the few transcription factors they require [23]. Type I IFNs are produced mainly when PRRs on the cell surface or in the cytoplasm of most cell types are stimulated by PAMPs [24,25,26]. Among the PRRs are Toll-like receptors (TLRs), NOD-like receptors (NLRs), and RIG-like receptors (RLRs). In the TLR pathway, Toll/IL-1R domain-containing adaptor-inducing IFN-beta (TRIF) or myeloid differentiation factor-88 (MyD88) components are recruited to the activated receptor after the receptors receive a stimulus from viral ds/ss DNA/RNA [27]. Then, receptor-associated kinases such as TAK1 and tank-binding kinase 1 (TBK1) induce phosphorylation of key transcription factors such as IFN regulatory factor 3 (IRF3), IFN regulatory factor 7 (IRF7), and nuclear factor kappa B (NF-κB), which are recruited to the promoter region of type I IFN genes to induce their transcriptions. In the RLR pathway, the retinoic acid-inducible gene (RIG-I), melanoma differentiation-associated protein 5 (MDA5), and laboratory of genetics and physiology 2 (LGP2) are activated after detecting viral RNA. LSm14A, a member of the LSm family, is a sensor of viral RNA and DNA that regulates the IFN response through RIG-I, which plays an important role in initiating IFN-β induction during the early stages of viral infection [28]. TRIM25 acts as an E3-ubiquitin ligase, whose RING structural domain interacts with RIG-I to mediate the caspase activation of RIG-I and interaction between its recruitment domain (CARD) and mitochondrial antiviral signaling protein (MAVS) [29]. RLR activates MAVS through aggregation, thereby activating IRF3, IRF7, and NF-κB, finally initiating type I IFN expression [30].

IFN then acts on specific cellular receptors to enhance the expression of hundreds of different genes collectively known as ISGs, including PKR, ISG15, ISG20, etc., thereby inducing an antiviral state [31,32,33,34]. Type I IFNs will bind to their specific receptors (a heterodimeric cell surface transmembrane receptor composed of IFNAR1 and IFNAR2 subunits) [35]. Then, the receptors rely on related Janus kinases (JAKs), such as TYK2 and JAK1 for signaling [35,36]. Activated JAKs phosphorylate specific tyrosine residues on the receptors, which dock with STAT1 and STAT2 [37], thereby phosphorylating them. Phosphorylated STAT1 and STAT2 bind to each other to form a stable heterodimer: the STAT1-STAT2 heterodimer. This dimer interacts with IRF9 to form the IRF9-STAT1-STAT2 heterodimer (ISGF3), which is translocated to the nucleus. ISGF3 induces the transcriptions of ISGs by activating the ISRE-containing promoter [38]. Additionally, type I IFNs and type II IFNs promote the formation of STAT1 dimer, termed the IFN-γ-activated factor (GAF), which is translocated to the nucleus and binds to the gamma-activated site (GAS) to induce ISGs expression [22,23,39].

**Figure 1:**
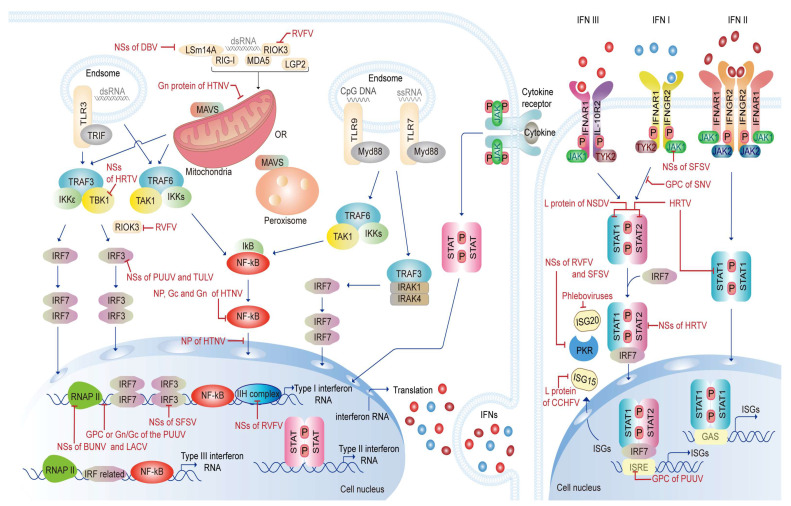
Anti-viral IFN response pathways and known suppression mechanisms of it in the order Bunyavirales.

Similar to type I IFNs, type III IFNs are induced by viral-derived PAMPs, but the molecular mechanism is not yet clear [39]. Induction of type III IFNs by the RLRs pathway is similar to that of type I IFNs. Different from type I IFNs, MAVS can be recruited to peroxisome and mitochondria, which is recruited to peroxisome and induces type III IFNs preferentially [40]. Type III IFNs have a similar signaling pattern to type I IFNs [41], which is achieved by binding to their receptor complexes, and signaling also occurs through the JAK-STAT signaling pathway. The receptor complexes consist of cell surface-specific IFNLR1 and IL-10R2 (Figure 1) [42].

IFN-γ is the sole member of type II IFN, which is produced primarily by antigen-activated T cells and NK cells. Additionally, macrophages secrete it under certain circumstances [43,44]. The antiviral effect of IFN-γ mostly relies on PKR, adenosine deaminase RNA specific-1 (ADAR-1), interferon-induced transmembrane proteins (IFITMs), and tripartite motif-containing proteins (TRIMs) that are induced by IFN-γ [45]. IFN-γ is induced by mitogens and cytokines such as IL-12, IL-15, IL-18, IL-27, and type I IFNs [46,47]. JAKs are activated when these cytokines bind to corresponding cell surface receptors, which in turn phosphorylates and activates STATs. Subsequently, the production and secretion of IFN-γ are promoted [47,48]. These STATs may be STAT1, STAT3, STAT4, or STAT5 [47]. The signaling pattern of type II IFNs is similar to type I IFNs and type III IFNs. The IFNGR complex is the type II IFN receptor. The difference is that IFN-γ signaling mainly activates GAF that targets GAS DNA sequences. Furthermore, IFN-γ also can activate ISGF3 [22,23,39] (Figure 1).

A. After virus invasion, the IFN pathway is activated to play an antiviral role. IFNs are categorized into three types, type I, type II, and type III. The PRRs include Toll-like receptors (TLRs), NOD-like receptors (NLRs), and RIG-like receptors (RLRs). In the TLR pathway, viral ds/ss DNA/RNA is recognized by TLRs on the endosome after the virus is broken down in the endosome. Then Toll/IL-1R domain-containing adaptor-inducing IFN-beta (TRIF) or myeloid differentiation factor-88 (MyD88) are recruited to the TLRs. TAK1 and TBK1 are activated to induce phosphorylation of IFN regulatory factor 3 (IRF3), IFN regulatory factor 7 (IRF7), and nuclear factor kappa B (NF-κB), which are recruited to the promoter region of type I IFN genes to induce their transcriptions. In the RLR pathway, the retinoic acid-inducible gene (RIG-I), melanoma differentiation-associated protein 5 (MDA5), and laboratory of genetics and physiology 2 (LGP2) are activated after detecting viral RNA. RLR activates mitochondrial antiviral signaling protein (MAVS) through aggregation, thereby activating IRF3, IRF7, and NF-κB, finally initiating type I IFN expression. LSm14A, a sensor of viral RNA and DNA, regulates the IFN response through RIG-I. TRIM25 interacts with RIG-I to mediate the activation of RIG-I and the interaction between RIG-I and MAVS. The generated type I IFNs will bind to their specific receptors. Then the receptors rely on related Janus kinases (JAKs), such as TYK2 and JAK1 for signaling, activated JAKs phosphorylate specific tyrosine residues on the receptors, which dock with STAT1 and STAT2, make them phosphorylated, and form a stable STAT1-STAT2 heterodimer. This dimer interacts with IRF9 to form the IRF9-STAT1-STAT2 heterodimer (ISGF3), which is translocated to the nucleus to induce the transcriptions of IFN-stimulated genes (ISGs) by activating the IFN-stimulated response element (ISRE)-containing promoter. Additionally, type I IFNs and type II IFNs promote the formation of STAT1 dimer, termed IFN-γ-activated factor (GAF), which is translocated to the nucleus and binds to the gamma-activated site (GAS) to induce ISGs expression. Cytokines can bind to corresponding cell surface receptors, make JAKs phosphorylation and activate STATs, then promote the production and secretion of type II IFN. The signaling pattern of type II IFNs is similar to type I IFNs. The differences are that the IFNGR is the type II IFN receptor and IFN-γ signaling mainly activates GAF that targets GAS. Induction of type III IFNs by the RLRs pathway is similar to that of type I IFNs. The difference is that MAVS can be recruited to peroxisome and mitochondria, which is recruited to peroxisome and induces type III IFNs preferentially. Type III IFNs have a similar signaling pattern to type I IFNs, achieved by binding to their receptor complexes (FNLR1 and IL-10R2).
B. In the interferon-producing pathway, viruses can act at different nodes to inhibit the antiviral effect of interferon. NSs of Dabie bandavirus (DBV) can interact and co-localize with LSm14A to inhibit the activation of RIG-I to reduce IRF3 phosphorylation and dimerization. Rift Valley fever virus (RVFV) can induce alternative splicing of RIOK3 transcript and then attenuate IFN response. Gn protein of hantaan virus (HTNV) can translocate to mitochondria and interact with mitochondrial Tu translation elongation factor (TUFM) to recruit LC3B and degrade MAVS. NSs of heartland virus (HRTV) can interact with TBK1 to block the binding of TBK1 to IRF3, thus inhibiting type I IFN induction. NSs of Puumala virus (PUUV)/Tula virus (TULV) can inhibit downstream factor IRF3 activity and IFN-β promoter. High-dose NP, Gc, and Gn of HTNV can inhibit NF-κB activation and interferon-stimulated gene expression and NP can sequester NF-κB in the cytoplasm and inhibit the activity of NF-κB. Bunyamwera orthobunyavirus (BUNV) NSs interact with MED8 to prevent the phosphorylation of RNA polymerase II (RNAP II). La Crosse virus (LACV) NSs degrade the IIo-borne RBP1 subunits by exploiting the cellular DNA damage. GPC or Gn/Gc of PUUV can inhibit the activation of IFN-β promoter. Sandfly fever Sicilian virus (SFSV) NSs can inhibit the induction of IFN by shielding the DNA-binding domain of IRF3. RVFV NSs can interfere with the formation of the transcription factor IIH complex. SFSV NSs can directly interact with JAK1 to inhibit STAT1 phosphorylation. GPC of Sin Nombre virus (SNV) can strongly inhibit the induction of IFN-β and the downstream amplification of the JAK-STAT pathway. The L protein of the Nairobi sheep disease virus (NSDV) can inhibit the phosphorylation of STAT1 and STAT2. HRTV can directly inhibit STAT1 without the help of viral protein and its NSs can interact and colocalize with STAT2 to specifically block the nuclear translocation of STAT2. RVFV NSs can trigger degradation of PKR and SFSV NSs can bind to eIF2B and maintain eIF2B activity against translational inhibition caused by PKR. Phleboviruses can largely escape the antiviral effect of ISG20. The L protein of Crimean–Congo hemorrhagic fever virus (CCHFV) can reduce ISG15-encoded ubiquitin and the conjugation of ubiquitin-like proteins with cellular proteins. GPC of PUUV can antagonize the promoter of ISRE on ISGs.

## 3. Suppressing IFN Response Mechanisms of Bunyavirales

The order Bunyavirales consists of RNA viruses, including more than 500 different viruses and strains, which are classified into fourteen families. These viruses utilize host cell proteins to facilitate their replication and transcription. The viral invasion of host cells enhances the transcriptional level of IFN, NF-κB, and inflammasome signaling pathways, inducing the expression of a large number of pro-inflammatory cytokines, and finally initiating the host antiviral innate immune response, the IFN response. The virulence of pathogenic bunyaviruses is directly related to the actions of viral virulence factors and their ability to counteract host immunity. Arthropod-borne viruses are distributed worldwide. Although some remain unnoticed or are associated with mild flu-like symptoms, many are significant human and veterinary pathogens that cause severe diseases such as arthritis, gastroenteritis, encephalitis, hemorrhagic fever, etc. Moreover, they lead to devastating economic losses due to reduced livestock productivity and high mortality rates. RNA gene segments of different bunyaviruses are heterogeneous, but most of them share similar characteristic molecular structures; that is, the presence of viral nonstructural proteins as interferon antagonists. In this context, we mainly discuss the strategies employed by bunyaviruses to evade the antiviral state induced by IFN in infected cells.

**Figure 2:**
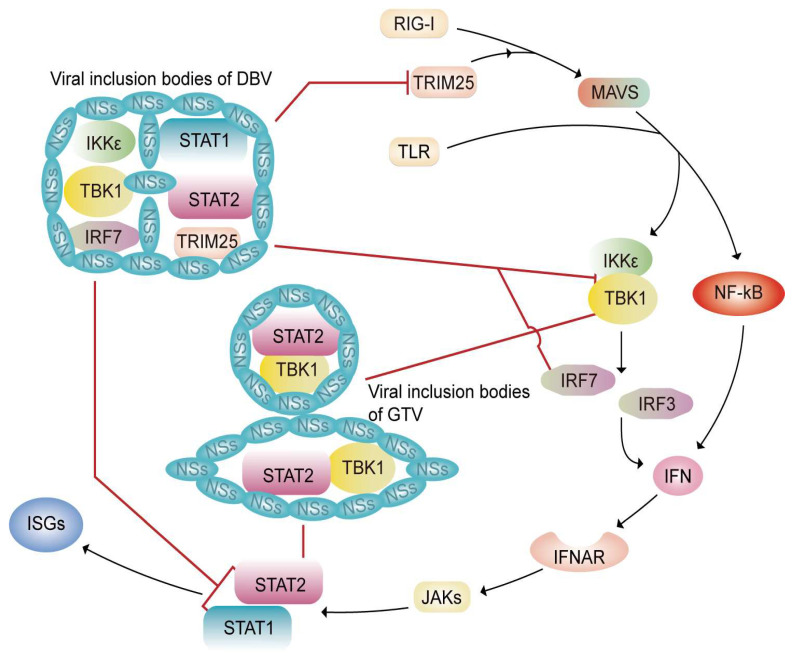
Model for DBV and GTV suppressing the IFN antiviral system by NS sequestration of key signaling molecules into IBs or FSs. After Dabie bandavirus (DBV) invades the host cells, TRIM25, tank-binding kinase 1 (TBK1), Ikkε, IFN regulatory factor 7 (IRF7), and STAT2, which are involved in the IFN signaling pathway, interact with DBV NSs and are captured into inclusion bodies (IBs), thereby inhibiting the IFN signaling pathway. TRIM25 is isolated to the viral IBs to inhibit TRIM25-mediated retinoic acid-inducible gene (RIG-I)-Ly-63-linked ubiquitination or activation. Reduction in intracellular free TBK1 and IKKε interferes with TBK1/IKKε- IFN regulatory factor 3 (IRF3)/IRF7 signaling. DBV NSs prevent the nuclear translocation of IRF7 by sequestering it to IBs. The phosphorylation and nuclear translocation of STAT2 are inhibited by IBs, which block type I IFN-stimulated JAK-STAT signaling. STAT1 may be tethered to the viral IBs that block its nuclear translocation, resulting in the inhibition of IFN signaling and IFN-stimulated genes (ISGs) expression. Likewise, NSs of Guertu virus (GTV) hijack TBK1 and STAT2 into IBs and filamentous structures (FSs) affecting the corresponding signal transduction.

DBV is a novel kind of bunyavirus. The IFN inhibitory response of DBV is primarily mediated by NSs and cytoplasmic structures called viral inclusion bodies (IBs) that they induce. NSs can directly interact with signaling molecules such as TRIM25, TBK1, Ikk, IRF7, and STAT2, and sequester them into the viral IBs, thereby inhibiting the IFN response. NS protein interacts with TRIM25 and tethers it to the viral IBs in order to inhibit TRIM25-mediated RIG-I-Ly-63-linked ubiquitination or activation, thus suppressing RLR-mediated antiviral signaling and IFN gene expression [49,50]. DBV NSs can effectively capture TBK1 and IKKε to IBs, interfering with TBK1/IKKε-IRF3/IRF7 signaling, which is closely associated with the amino acids serine 21 and leucine 23 in NSs [51,52]. DBV NSs can also sequester IRF7 and STAT2, but not STAT1 to IBs, thereby preventing STAT2 phosphorylation and nuclear translocation, eventually blocking type I IFN-stimulated JAK-STAT signaling and inhibiting the expression of ISGs [53,54]. However, there is a study showing that NSs can isolate both into viral IBs to impair STAT2 phosphorylation induced by IFN and the nuclear translocation of both STATs, leading to inhibition of the IFN-JAK-STAT signaling pathway and ISGs expression [55] (Figure 2). Additionally, LSm14A plays an important role in the early induction of IFN-beta during virus infection. Nevertheless, DBV NSs effectively inhibit IRF3 phosphorylation and dimerization by interacting and co-localizing with LSm14A. The second arginine in the conserved leucine-rich repeat domain (LRRD) at the N-terminus of NSs is crucial for its interaction with LSm14A [56].

Guertu virus (GTV) is a newly isolated potential highly pathogenic bunyavirus in China, with genetic relation to DBV and HRTV. In terms of GTV, its cytoplasmic structures antagonizing IFN response differ from DBV. GTV NSs (G-NSs) induce the formation of dense IBs and filamentous structures (FSs) in the cytoplasm. G-NSs interact with TBK1 and STAT2, thereby irreversibly isolating them into the viral IBs and FSs, finally inhibiting the phosphorylation, activation, and nuclear translocation of IRF3 [57] (Figure 2).

HRTV is a recently reclassified member of the genus Bandavirus, family Phenuiviridae of order Bunyavirales. Likewise, HRTV can antagonize the host’s innate immune response. However, it does not encode any viral protein but acts as a direct antagonist against STAT1, and it can reduce the activation of STAT2 and STAT1 induced by type I and type III IFNs excluding IFNγ [58]. The similarity in the mechanism of IFN response antagonism between HRTV and DBV lies in the fact that the NSs of both viruses can interact with TBK1 and then hinder the binding of TBK1 to its substrate IRF3, thus blocking IRF3 activation and cellular transcriptional induction, which depends on two conserved amino acids at positions 21 and 23 [52,59]. Additionally, both viral NS proteins can interact with STAT2 to prevent its nuclear translocation. The difference is that HRTV NSs do not interact with STAT1 and do not affect the nuclear translocation of STAT1 [58]. Yet now there is no research indicating that HRTV NSs can induce unique cytoplasmic structures such as viral inclusion bodies and filamentous structures.

Similar to DBV, GTV, and HRTV, Nairobi sheep disease virus (NSDV) can also inhibit the production of IFN by interfering with STAT1 and STAT2. NSDV is another member of the genus Orthonairovirus in the family Nairoviridae, which is a tick-borne zoonotic pathogen genetically related to the human-pathogenic CCHFV. NSDV can lead to severe and often fatal hemorrhagic gastroenteritis. There is evidence that the L protein of NSDV can inhibit the phosphorylation of STAT1 and STAT2 in Vero cells, particularly in response to I and II type IFN stimulation, with STAT1 being the primary target and this inhibitory effect is related to the enzymatic activity of the N-terminal OTU domain of the L protein [60].

Rift Valley fever virus (RVFV) is also a member of the order Bunyavirales, belonging to the Phlebovirus genus, family Phenuiviridae. It caused periodic outbreaks in livestock and humans in African and Middle Eastern countries. RVFV NS is a major virulence factor that disrupts the host-innate immune response and antagonizes IFN [61]. The NSs protein of RVFV can inhibit the transcription of type I IFN-β gene, but strains with intact NS protein can still activate the dimerization and nuclear translocation of RIG-I, IRF3, and nuclear translocation of NF-κB, which suggests that NSs protein inhibits the up-regulation of downstream IFN-β promoters activated by transcription factors to evade host innate immunity [62]. RVFV NSs can interact with two transcription factors, SAP30 and YY1, that regulate IFN-β to interfere with the formation of the transcription factor IIH complex, degrade the p62 subunit, and sequester the p44 subunit, which finally suppresses the host’s IFN-β gene transcription and can trigger specific degradation of PKR through proteasomal degradation [63,64,65]. A significant portion of the antiviral activity of IFN against RVFV was attributed to PKR. The NSs can induce the degradation of PKR, thereby preventing the phosphorylation of eIF2α in cells, allowing the virus to efficiently translate and replicate [66]. RIO kinase 3 (RIOK3) is a protein kinase that facilitates the activation of MDA5 and IRF3 in the process of IFN-β production [67,68]. Experiments show that RVFV infection induces selective splicing of the RIOK3 transcript, which weakens the IFN response [69]. Furthermore, Barski proposed that the filament arrangement of the NS’s core structural domain in the crystal may reveal the molecular basis of how the virulence factor NS assembles in the cell nucleus, which is crucial for a fundamental understanding of RVFV’s interference with the IFN immune response [70].

The NSs of Sandfly fever Sicilian virus (SFSV) play an important role in suppressing interferon responses. The SFSV can not only inhibit IRF3 and JAK-STAT pathway, but also inhibit PKR as RVFV. SFSV is one of the most common members of the genus Phlebovirus, family Phenuiviridae. Its NSs can inhibit IFN induction by blocking the IRF3 DNA-binding domain, thereby interfering with the RIG-I-TBK1-IRF3 signaling pathway [71]. SFSV NSs can also directly interact with JAK1 to inhibit STAT1 phosphorylation and nuclear translocation, thereby suppressing the signal transduction of the type I IFN [72]. Its NSs tightly bind to eIF2B to block the binding of eIF2-p to eIF2B, thereby maintaining the activity of eIF2B to prevent translation inhibition caused by PKR, eventually maintaining the synthesis of viral proteins [73].

Hantaan virus (HTNV) not only acts on NF-κB similar to RVFV but also acts on MAVS to suppress IFN. Moreover, the glycoprotein N (Gn) and nucleocapsid protein (NP) of HTNV play an essential role in IFN suppression. HTNV is the pathogen responsible for Korean hemorrhagic fever, which belongs to the orthohantavirus genus (order Bunyavirales, family Hantaviridae). HTNV can induce hemorrhagic fever with renal syndrome characterized by endothelial dysfunction, as well as hantavirus pulmonary syndrome, both of which have high fatality rates. HTNV infection can suppress the antiviral effects of all IFNs (including type III IFN). NP can sequester NF-κB in the cytoplasm, inhibiting tumor necrosis factor-alpha (TNF-α) and interfering with host innate immune responses [74]. Additionally, NP can enhance the activity of the miR-146a promoter, promoting miR-146a expression and its negative regulatory effect on the NF-κB pathway [75]. At high doses, both NP and envelope proteins (Gn and Gc) can inhibit NF-κB activation and the expression of IFN-stimulated genes, thus suppressing the IFN response [76]. In the early stages of infection, HTNV induces complete mitophagy, followed by incomplete mitophagy in later stages and these responses involve the viral Gn and NP. The viral Gn in infected cells can translocate to the mitochondria and interact with TUFM, promoting mitophagy by recruiting LC3B. Then the degradation of MAVS is promoted, finally suppressing the type I IFN response. The viral NP competes with Gn for binding to LC3B and interacts with SNAP29 to disturb the autophagic degradation of Gn [77]. Consequently, both Gn and NP of HTNV can inhibit IFN response by degrading MAVS.

Puumala virus (PUUV), Tula virus (TULV), and Sin Nombre virus (SNV) are the other three members of the orthohantavirus genus (order Bunyavirales, family Hantaviridae). The glycoprotein precursor (GPC) is important for the IFN suppression of them. The GPC or its cleavage product Gn/Gc of PUUV can inhibit the activation of IFN-β promoter triggered by RIG-I. Furthermore, the GPC of PUUV and TULV can antagonize the promoter of IFN-stimulated response element (ISRE) on ISGs [78]. Likewise, the GPC of SNV strongly inhibits IFN-β induction and signaling transduction by suppressing downstream signaling of the JAK-STAT pathway [79]. The functional NSs of both PUUV and TULV can inhibit the activity of downstream factor IRF3 and IFN-β promoter [80].

The similarity between Bunyamwera orthobunyavirus (BUNV) and LACV lies in that the NSs of both can suppress the induction of IFN by affecting RNAP II. BUNV is the prototype of the genus orthobunyavirus (Bunyavirales: Peribunyaviridae), which poses a significant threat to human health and food security. LACV is the major cause of pediatric arboviral encephalitis in the United States and LACV encephalitis can result in learning and memory impairments, with over 80% of reported cases of neurological diseases occurring in children. The N-terminal and C-terminal regions of BUNV NSs play a critical role in countering the host antiviral response [81,82,83]. The C-terminal region of BUNV NSs interacts with cellular mediator protein MED8 to prevent the phosphorylation of serine 2 in the C-terminal domain (CTD) of RNAP II, thereby interfering with IFN synthesis of the mammalian host [82]. NSs from BUNV and RVFV interact with two host cell factors, MED8 and p44, to inhibit RNAP II. However, LACV NSs employ a distinct mechanism to inhibit or degrade RNAP II. In LACV-infected cells, NSs degrade the IIo-borne RBP1 subunits by exploiting the cellular DNA damage response pathway [84]. Research suggested that the E3 ubiquitin ligase elongin C subunit of LACV acts as an NS co-factor in RBP1 degradation to block RIG-I-induced IFN production and inhibit the synthesis of IFN-related mRNA [85]. Tacaiuma virus (TCMV), another member of the genus Orthobunyavirus in the family Peribunyaviridae, causes an acute febrile illness accompanied by muscle and joint pain [86], whose pathogenic mechanism does not involve NSs.

ISG20 and ISG15 are the other two key points of IFN suppression in some members of the order Bunyavirales infections. ISG20 is an anti-bunyavirus factor, and its anti-bunyavirus activity depends on its functional RNase activity. ISG20 has strong antiviral activity against most viruses from the Peribunyaviridae, Hantavirus, and Nairovirus genera, but it does not affect Phleboviruses [87]. This suggests that the ability of Phleboviruses to escape ISG20 may affect their pathogenesis. CCHFV belongs to the genus Orthonairovirus in the family Nairoviridae of the order Bunyavirales. Earlier research discovered that the L protein of CCHFV reduces ISG15-encoded ubiquitin and the conjugation of ubiquitin-like proteins with cellular proteins, and then interferes with IFN induction, which is related to the N-terminal OTU domain of the L protein [60]. Meanwhile, there is evidence that the NSDV negatively regulates the IFN induction by encoding an OTU family protease [88].

In summary, host cells can sense viral infections and activate the innate immune system to inhibit viral replication and propagation. When PAMPs are recognized by PRRs within cells, numerous signaling cascades are activated, thereby leading to the production of IFNs. IFNs establish an antiviral state by inducing the expression of hundreds of ISGs in an autocrine and/or paracrine manner. The IFN response induced after bunyavirus infections can restrict the infection at various steps of the viral life cycle, including viral entry, genome transcription and replication, and viral particle exocytosis. The main processes of the IFN response include TLR-dependent signaling, MDA5 and RIG-I-dependent signaling, IFN antiviral signaling transduction, and IFN-inducible transcriptions. Bunyaviruses can counteract this antiviral response by avoiding cellular recognition of PAMPs, inhibiting IFN production, or interfering with IFN-mediated antiviral responses (Figure 1 and Figure 2). Furthermore, the key targets involved in this counteraction include RLRs, TBK1, IRF3, and JAK, among other immune molecules (Table 1). Therefore, it may serve as a potential measure in the treatment of bunyavirus infections to target directly or indirectly these key immune molecules.

## 4. Potential Treatment Options for IFN Suppression

IFN response plays a crucial role in the development of various diseases, and numerous studies explored the impact of regulating its key targets on diseases. Some of these studies were already applied clinically in antiviral treatments. However, in terms of bunyavirus infections, specific therapeutic research is lacking. Nevertheless, certain drugs, compounds, or methods associated with IFN response regulation might possess potential antiviral effects against bunyaviruses, which are expected to be used in the treatment of bunyavirus infections. Based on the key IFN pathway nodes and viral proteins of the suppression of IFN response in bunyavirus infections, this article reviews drugs, compounds, and methods closely related to their regulation and analyzes their clinical applications, particularly in the context of virus infections (Table 2). These treatments directly or indirectly influence IFN response and its antiviral signaling, representing potential therapeutic strategies for bunyavirus infections.

### 4.1. Key Targets of the IFN Pathway

#### 4.1.1. RLR

RLRs are crucial sensors in viral infections and important inducers of type I IFNs and other antiviral immune mediators. This protein family includes three members: RIG-I, MDA5, and LGP2. Among these, RIG-I and MDA5 are important inducers of type I IFN [179]. However, some viruses within the order Bunyavirales, such as DBV and RVFV, can suppress the IFN induction by impacting their activity. There are experimental and clinical studies showing that RLRs can be activated by synthetic RNA, oncolytic viruses, viral mimicry, and radiochemotherapy, leading to the expression of RLR-mediated IFNs and ISGs [180], which is essential for antiviral immunity in the host.

##### RIG-I and MDA5

Polyinosinic-polycytidylic acid (polyI:C) is a synthetic analog of double-stranded RNA as a viral mimic, which also is an agonist for TLR3 and RLR. It enhances RLR signaling, thereby strengthening RLR-mediated IFN responses to exhibit antiviral effects. Research demonstrated that polyI:C-induced IFN responses have a protective effect against mouse Mengo virus infection [89]. Furthermore, a recombinant trimeric spike protein vaccine containing PIKA, a stabilized derivative of polyI:C, as an adjuvant was proven to be effective against COVID-19 [91]. Another vaccine formulation containing polyI:C, innate defense regulator peptide, and polyphosphazine (ΔF/TriAdj) effectively immunizes newborns against respiratory syncytial virus (RSV) [92]. Thus, polyI:C might be a vital potential therapeutic compound and vaccine adjuvant for bunyaviruses. BO-112, a nanocomposite formulation of Poly I:C, and polyethyleneimine, can induce apoptosis in tumor cells displaying immunogenic cell death features [90]. It is capable of stimulating IFN response by polyI:C, thereby exerting its antiviral effects.

Mesenchymal stromal cells (MSCs) derived from bone marrow (BM), adipose tissue (AT), Wharton’s jelly (WJ), and foreskin (FSK) have functional RIG-I and MDA-5 receptors. Upon receptor activation, IFN production is induced through TBK1/IKK-ε and IRF7 phosphorylation. Consequently, IFN’s antiviral immune protective effect is enhanced [94]. Currently, MSCs made significant breakthroughs in addressing hematologic disorders, cardiovascular diseases, cirrhosis, neurological disorders, partial meniscectomy injury repair, and autoimmune diseases [93,95]. There are numerous completed or ongoing clinical trials exploring MSCs’ preventive or therapeutic effects against HIV and hepatitis B virus (HBV) infections [97]. A series of phase 2 clinical trials showed that MSCs can be used to treat patients with severe COVID-19 patients [96,98]. Notably, MSCs can enhance virus-induced IFN responses, and are widely used in clinical antiviral treatment research, showing potential in bunyavirus infections treatment.

##### RIG-I

The U.S. Food and Drug Administration (FDA)-approved oral drug nitazoxanide (NTZ) targeting RIG-I and antiviral phosphatases GADD34 can increase the activities of RLR, mitochondrial antiviral signaling protein, IRF3, and IFN, which can also induce the transcription of GADD34 [101]. Currently, nitazoxanide is FDA-approved for the treatment of diarrhea caused by Cryptosporidium infection and is well-tolerated. Additionally, clinical trials indicated that NTZ reduces the duration of symptoms for influenza [100], viral gastroenteritis [102], and rotavirus (RV)-induced diarrhea [99]. It is also a potential oral therapy for the Ebola virus (EBOV) [101]. Nitazoxanide can enhance IFN antiviral immune responses, finally exhibiting antiviral effects, which represents a potential drug for treating bunyavirus infections.

Another drug approved by the FDA is acitretin, which induces RIG-I expression and enhances RIG-I signaling in vitro, thereby stimulating RIG-I-dependent IFN cascade reactions and inducing preferential apoptosis of HIV-infected cells. It is a potential drug to eliminate reactivating cells in latent HIV reservoirs [104]. Research showed that acitretin selectively inhibits BK polyomavirus (BKPyV) replication in primary human cells in nephropathy and hemorrhagic cystitis [103]. Combined immunotherapy using systemic acitretin and diphenylcyclopropenone effectively treats recalcitrant viral warts caused by human papillomavirus (HPV) infections [105]. Acitretin promotes RIG-I-dependent IFN response and antiviral effects. Therefore, acitretin might be a potential drug for treating bunyavirus infections in the future.

Salidroside is a prolyl endopeptidase inhibitor that exerts antiviral activity against dengue virus (DENV) by increasing the expression of RIG-I, thereby enhancing downstream signal cascades such as upregulation of IRF-3 and IRF-7, eventually stimulating IFN-mediated innate antiviral immunity. Additionally, it inhibits virus protein synthesis by increasing the expression of PKR and P-eIF2α while suppressing NF-κB expression [107]. Notably, salidroside has highly effective antiviral effects. Salidroside used as an adaptogen in traditional Chinese medicine and possesses a wide range of pharmacological characteristics, including anti-hypoxia, anti-aging, anti-cancer, anti-inflammatory, antioxidant, antiviral, immune-stimulating activity, anti-diabetic, etc. [109]. Nowadays, it can not only be isolated from Rhodiola species, but also be obtained through synthetic pathways [109]. Animal experiments show that salidroside exhibits antiviral activity against coxsackievirus B3 (CVB3) [106] and Influenza A virus (IAV) [108], suggesting its potential as a promising therapeutic agent for bunyavirus infections.

RNA agonists of RIG-I can enhance the IFN antiviral immune response. RNA agonists of RIG-I are widely used in preclinical and clinical studies for cancer and viral therapy. Some of the identified RNA agonists of RIG-I include 3p10LG9 [113], 5′-ppp RNA [115], 5′-ppp siRNA [111], 3p-siRNA-MDR1 [110], SLR14 [114], etc. RNA agonists of RLR can be delivered in vivo through nanoparticles, extracellular vesicles, and CAR-T cells [180]. For instance, in vitro studies demonstrated the practicality of biocompatible gold nanorod GNR-5′PPP-ssRNA, a nano-complexes as an antiviral strategy against IAV [112]. Additionally, it was indicated that a novel small dsRNA hairpin, 3p10LG9, can control DENV infection [113]. Another study suggests that 5′-ppp RNA effectively blocks herpes simplex virus 1 (HSV-1) infection in vitro and in vivo through a STING-dependent mechanism [181]. Furthermore, SLR14, a stem-loop RNA, exhibits antiviral capabilities for SARS-CoV-2 infection in a mouse model [114]. Therefore, RNA agonists of RIG-I possess broad-spectrum antiviral activity and hold potential therapeutic value in treating bunyavirus infections.

Ionizing radiation (IR) stimulates cytoplasmic RIG-I to bind with small endogenous non-coding RNAs (sncRNAs), thereby inducing IFN production. Combined therapy with RLR agonists and ionizing radiation can have a radiosensitizing effect [117]. Some studies suggested that ionizing radiation has effects against the zika virus (ZIKV), influenza virus, and poliovirus. With certain intensities of X-rays, gamma rays, or UVC light, the replication and proliferation of SARS-CoV-2 in the lungs of COVID-19 patients can be non-invasively inhibited [122]. Another study showed that the combination of UV-4B and IFN-α enhances the antiviral activity against Dengue virus [116]. Similar to the DNA-damaging effect of radiation therapy, treatment with DNA-damaging agents such as doxorubicin [120], etoposide [119], teniposide [118], and oxaliplatin [121] can induce type I IFN responses and activate dendritic cells and CD8 T cells. Hence, radiation therapy and the application of DNA-damaging agents can strengthen type I IFN-mediated antiviral effects, which potentially serve as the therapeutic strategy for bunyavirus infections.

##### MDA5

DNA demethylation agents can simulate viral infection by inducing dsRNA that is used in cancer immunotherapy. This viral mimicry leads to an antiviral response mediated by the cytoplasmic pattern recognition receptor MDA5, followed by MAVS activation, IRF7 nuclear translocation, and upregulation of type III IFN and ISGs [125]. Finally, it enhances the IFN response triggered by viruses and represents a potential strategy for treating bunyavirus infections. DNA methyltransferase inhibitors (DNMTis) are a type of DNA demethylation agent, including 5-azacytidine (AZA) and 5-aza-2′-deoxycytidine (DAC), which are widely used in the treatment of hematological diseases [123,124]. The new oral DNMTi ASTX727, consisting of decitabine and the cytidine deaminase (CDA) inhibitor cedazuridine, was approved by the FDA in 2020 for the treatment of high-risk MDS and CMML. CC-486, an oral formulation of Aza, received FDA approval for the treatment of AML in elderly patients who achieved first remission with intensive chemotherapy [123]. Currently, DNA methyltransferase inhibitors are successfully applied in clinical hematologic malignancies, but their potential in antiviral therapy requires further exploration and research.

#### 4.1.2. TBK1

As described above, TBK1 is an important target in the suppression of IFN response for viruses belonging to the family Phenuiviridae of order Bunyavirales, such as DBV, GTV, and HRTV. Currently, there are numerous studies on TBK1 inhibitors in tumor therapy, but there is still a lack of research on its activators and their application in antiviral therapy. Lidocaine can promote the phosphorylation of TBK1 and IRF7, as well as the activation of JNK and downstream factors such as c-Jun through the TBK1-IRF7 and JNK-AP1 signaling pathways, and then it upregulates IFNα4 via SHP2 to have antiviral potential. Moreover, lidocaine also has potential adjunctive effects in the treatment of IAV and even SARS-CoV-2 infections [126]. Therefore, it is speculated that lidocaine has antiviral efficacy against bunyavirus through upregulation of the antiviral IFN response. Additionally, research shows that lidocaine has an anti-inflammatory effect by inhibiting the activation of NF-κB and p38 MAPK, which is a new therapeutic agent for the treatment of allergic rhinitis (AR) [182]. As a consequence, we presume that lidocaine has a potential therapeutic effect on cytokine storms triggered by bunyavirus. Further studies are required to investigate the potential of lidocaine to treat bunyavirus infections.

#### 4.1.3. IRF3

PUUV, TULV, and SFSV can inhibit the activity of IRF3, thereby reducing the IFN induction. So, the following compounds targeting IRF3 may be applied to the treatment of bunyavirus in the future.

Isoflavone and isoflavone-like compounds are potential therapeutic agents for bunyavirus infections. Isoflavone compounds belong to the class of flavonoids and serve as specific activators of the innate immune signaling pathway, leading to the activation and nuclear translocation of IRF3, eventually demonstrating high antiviral activity against HCV and influenza virus. KIN 101, an isoflavone compound, exhibits broad-spectrum antiviral activity against DNA and RNA viruses [127]. Isoflavone and isoflavone-like compounds showed antiviral properties both in vitro and in vivo. Among them, genistein, a soy-derived isoflavone, was extensively studied for its antiviral activity and was proven to inhibit the infectivity of enveloped or non-enveloped viruses, as well as single-stranded or double-stranded RNA or DNA viruses [128].

Previous research found that the hydroxyquinoline family compounds including KIN1400 and its derivatives, KIN1408 and KIN1409, possess broad-spectrum antiviral activity. These compounds selectively activate IRF3 to promote cellular IFN response, which demonstrates their antiviral efficacy. They can prevent or treat various RNA virus infections in cell cultures, such as West Nile virus (WNV), DENV, HCV, IAV, RSV, Nipah virus (NiV), Lassa virus (LASV), and EBOV [129]. Briefly, small molecules of the hydroxyquinoline family, such as KIN1400 and its derivatives KIN1408 and KIN1409, may exhibit activity against bunyavirus by strengthening the IFN antiviral effect induced by bunyavirus.

Additionally, vaccinia virus (VACV) and bunyavirus share similarities in their ability to inhibit IFN antiviral responses. Research designed and indicated that a recombinant VACV vaccine that expresses IRF-3 in mice induces a robust protective immune response against lethal VACV in mice [183]. This finding is significant for the development of bunyavirus vaccines.

#### 4.1.4. Others

Additionally, besides directly targeting key points in the IFN response induction and antiviral signaling as discussed above, there are several indirectly targeted therapy strategies with significant clinical value. While some of the directly targeted drugs or strategies are still in the pre-clinical research stage and require further exploration for their clinical application, some indirectly targeted drugs are widely used in clinical settings and were suggested to be closely associated with various viral infections. These indirect strategies that target IFN induction and its antiviral signaling pathway hold promise as effective measures in the treatment of bunyavirus infections. The following will discuss and summarize these potential therapeutic strategies of indirect targeting one by one.

The p97 complex promotes the proteasomal degradation of RIG-I, thereby reducing the antiviral innate immune response. Disruption of the p97 complex enhances antiviral signal transduction of RIG-I. Furthermore, P97 activity was proven to be crucial for the replication of viruses such as poliovirus, HSV, cytomegalovirus (CMV), influenza virus, etc. [130]. Currently, there are many p97 inhibitors including active sites and allosteric inhibitors. Although the drug clotrimazole was identified as a p97 inhibitor, its antiviral properties were not demonstrated [130,131]. In conclusion, it is a potential indirectly targeted therapy approach to target the p97 complex for treating bunyavirus infections. Further, p97 inhibitors along with clotrimazole may be used for the treatment of bunyavirus infections.

Mounting evidence shows that TBK1 plays an important role in the mechanism of IFN suppression in bunyavirus infections. There is a substantial amount of domestic and international research on estrogen-related receptor alpha (ERRα), especially its anti-tumor effects. Meanwhile, some studies indicated that ERRα can inhibit the induction of type I IFN, primarily through its interaction with TBK1 [133]. Additionally, it was suggested that ERRα is a potential target for treating human cytomegalovirus infection [134]. Therefore, targeting ERRα indirectly to modulate IFN induction by reducing ERRα activity can enhance the antiviral IFN response, which is expected to be used for controlling bunyavirus infections. It was discovered that XCT790 is a potent ERRα inverse agonist with antiviral efficacy, which is demonstrated to be effective against VSV, Newcastle disease virus (NDV), HSV, and HBV by regulating the IFN response [133,141]. Moreover, compounds such as N-[(2Z)-3-(4,5-dihydro-1,3-thiazol-2-yl)-1,3-thiazolidin-2-ylidene]-5H dibenzo[a,-d] [7]annulen-5-amine [132], kaempferol [137], cyclohexylmethyl-(1-p-tolyl-1H-indol-3-ylmethyl)-amine [135], LingH2-10 [139], naringenin [138], and SR16388 [140] were all identified to inhibit ERRα activity. Furthermore, research identified five anticancer drugs and nine pesticides capable of inhibiting ERRα activity [136]. Briefly, numerous drugs and compounds were identified as potential ERRα inhibitors with promising prospects for the treatment of bunyavirus infections that hold significant research value.

Just as mentioned above, IRF3 is a critical target for the mechanisms of IFN suppression in bunyavirus infections. The deficiency of 7-dehydrocholesterol reductase (DHCR7) or treatment with natural product 7-dehydrocholesterol (7-DHC) can specifically promote the phosphorylation of IRF3, thereby enhancing the production of type I IFN in macrophages [149]. Notably, DHCR7 deficiency and DHCR7 inhibitors such as AY9944 and the chemotherapeutic drug Tamoxifen promote the clearance of various viruses both in vitro and in vivo. It was shown that they can inhibit ZIKV, vesicular stomatitis virus (VSV), HCV, HSV, and SARS-CoV-2 infections [146,147,148,149]. Therefore, DHCR7 inhibitors and 7-DHC can enhance the innate antiviral immune response triggered by viral infections and may be used in the treatment of bunyavirus infections. There were some DHCR7 inhibitors identified among existing drugs, including Tamoxifen [142] and antipsychotic drugs such as haloperidol, aripiprazole, cariprazine, and trazodone [143,144]. Likewise, small molecules such as AY9944 and BM15.766 were confirmed to effectively inhibit DHCR7 [145,150]. Overall, these DHCR7 inhibitors hold potential as drugs for treating bunyavirus infections by indirectly targeting IRF3.

The bunyaviruses SNV and SFSV inhibit IFN signaling by interfering with the JAK-STAT pathway. In the JAK-STAT pathway, there is a suppressor of cytokine signaling called SOCS. SOCS-1 and SOCS-3 are the main members of the SOCS protein family, but they act differently. SOCS-1 directly binds to the JAK kinase domain to inhibit phosphorylation, while SOCS-3 binds directly to JAK kinases and cytokine receptors to inhibit JAK-STAT signaling [184]. SOCS1 and SOCS3 are intrinsic virulence factors for some virus infections, such as influenza A virus, encephalomyocarditis virus, HSV-1, vaccinia virus (VV), DENV, Zika virus, WNV, and EBOV [154]. Taken together, SOCS antagonists can serve as a method for treating these viral infections. Furthermore, research developed a small peptide antagonist of SOCS-1 called pJAK2 (1001–1013), which can prevent HSV-1 [152], IAV, VV [153], and encephalomyocarditis virus (EMCV) infections and inhibit their replication, demonstrating broad antiviral activity [151]. Consequently, it is feasible to target SOCS proteins indirectly to regulate JAK-STAT signaling in diseases. SOCS antagonists can enhance JAK-STAT signaling and then strengthen the IFN response to achieve antiviral effects. SOCS proteins represent potential targets for the treatment of bunyavirus infections, and SOCS antagonists such as pJAK2 (1001–1013) are potential compounds for treating bunyavirus infections.

MEK regulates the type I IFN response of human primary airway epithelial cells. There is evidence that MEK inhibitors (MEKi) can strengthen the type I IFN response after infecting with respiratory viruses rhinovirus (RV2) or respiratory syncytial virus (RSVA2) mainly by promoting the expression of IFN-β and the translation of ISGs [156]. In addition, trametinib was approved as a MEKi that inhibits the induction of IFN-β and then effectively blocks the replication of various subtypes of influenza A viruses in vitro and in vivo [155,157]. Therefore, trametinib, the MEKi, is expected to be used for bunyavirus infections by enhancing the type I IFN response.

### 4.2. Targeting Viral Proteins

#### 4.2.1. NSs

NSs are essential for many bunyaviruses (BUNV, LACV, DBV, RVFV, SFSV, etc.) to inhibit IFN response and mediate immune evasion. While there is currently a lack of research on therapeutic strategies targeting bunyavirus NSs, numerous studies demonstrated the potential value of using DENV NS1 as a therapeutic target and candidate vaccine. The approaches and methods of these studies hold significant insights and references for exploring antiviral treatment strategies targeting bunyavirus NSs. A study investigated the drug-like properties, binding affinity, interaction of plant compounds and DENV NS1 protein, and the interaction of NS1 and TLR4 through pharmacokinetics, molecular docking, binding affinity, interaction, and molecular dynamics. They found that three plant compounds named rutin, myricetin 3-rhamnoside, and kaempferol 3-(2″-rhamnosylrutinoside) bind to the critical amino acid residue ASN130 at the active site of the NS1 protein, thus improving platelet reduction in DENV-infected patients by disrupting the interaction of NS1 and TLR4 [185]. Another molecular docking-based study discovered that plant flavonoids can also bind to the ASN130 glycosylation site of DENV NS1 to potentially inhibit virus replication. These flavonoids are hopeful to be antiviral drugs for DENV infection [186]. Furthermore, there is evidence that anti-NS1 antibodies can serve as specific therapeutic drugs for dengue virus infection to block NS1-induced pathogenic effects both in vitro and in vivo, which reduce virus replication and lower mortality and morbidity in different mouse models of DENV infection [187,188]. NS1 is a candidate vaccine for DENV infection, as it can trigger both cellular and humoral immunity. Studies indicated that the mice inoculated with DENV NS1 by different methods can possess protective immune responses against DENV infection [189,190,191].

RNA interference (RNAi) is a method used to silence viral genes, and small interfering RNA (siRNA) was demonstrated as a potential therapeutic strategy against viral infections [161]. The siRNA that specifically targets the non-structural protein genes for various viruses such as IAV [158], porcine reproductive and respiratory syndrome virus (PRRSV) [160], WNV [159], and SARS-CoV-2 [162] can reduce viral titers, thereby inhibiting viral infections. In terms of bunyaviruses, there is a study indicating that RNAi activators have potential therapeutic benefits against RVFV infections. In a cell culture model of RVFV replication, short hairpin RNAs (shRNA) targeting the N gene reduced intracellular nucleocapsid protein concentration and viral replication, while shRNA targeting the NSs gene reduced NSs protein concentration, thereby alleviating NS-mediated cytotoxicity [192]. However, there is evidence that LACV, a member of the California serogroup of orthobunyaviruses, may have the capability to inhibit RNAi [193,194]. Therefore, RNAi-based viral gene silencing may be ineffective against LACV infection. In summary, siRNA targeting the NS gene of bunyaviruses can reduce NS expression, thereby inhibiting its antagonistic effect on IFN, which can provide antiviral benefits in bunyaviruses excluding LACV infection.

#### 4.2.2. NP

In the interference mechanism of IFN suppression in HTNV, NP plays a crucial role. NP can inhibit the NF-κB activity in the IFN antiviral pathway through various pathways. Therefore, targeting HTNV’s NP can counteract the virus’s immune escape mechanisms, presenting a potential strategy for treating HTNV infections. Various antiviral treatment approaches targeting the viral NP were extensively explored.

For instance, Sharifi et al. employed virtual screening through docking followed by molecular dynamics and discovered that doxycycline and minocycline are potential inhibitors of CCHFV NP [163]. Additionally, IAV’s NP is a critical target for antiviral therapy. Compounds such as curcumin [166] and naproxen [168] inhibit the NP-RNA binding required for IAV NP function, serving as novel antiviral drugs against IAV. Curcumin derivatives, such as tetramethylcurcumin, also exhibit inhibitory effects against IAV [195]. Furthermore, curcumin and its derivatives control EBOV infection by targeting NP [164] and demonstrate broad-spectrum antiviral activity against HIV, DENV, HSV-2, and other viruses [165]. Similarly, naproxen was found to target the NP of SARS-CoV-2, exhibiting antiviral properties [167]. Therefore, curcumin and naproxen, especially curcumin, may be potential candidates for HTNV treatment, warranting further research for validation. Compound RK424 [169] demonstrated effective antiviral activity against IAV by inhibiting NP’s RNA binding, oligomerization, and nuclear export functions, and validated in vitro and mouse models.

RNAi targeting the NS gene and RNAi targeting the NP gene through siRNA and shRNA provide a direct and rapid treatment for viral infections, proving to be effective against various viruses such as human metapneumovirus (hMPV) [171], rabies virus [170], Borna disease virus (BoDV-1) [173], and IAV [172]], which is a potential measure to treat HTNV infection. Moreover, NP-specific monoclonal antibodies (mAbs) were extensively developed for antiviral therapy against IAV [174]. Antisense oligonucleotides designed for the NP gene’s RNA binding region inhibit the replication of highly pathogenic avian influenza virus (AIV) H5N1, representing a novel therapeutic candidate [175]. These approaches offer new avenues for the treatment of HTNV infections.

#### 4.2.3. GPC

GPC participates in IFN suppression in PUUV and SNV and is a potential target for antiviral therapy. Research indicates that some antiviral treatments act by targeting GPC. Trametinib, for example, inhibits GPC-mediated membrane fusion by targeting the transmembrane (TM) domain of Lujo virus (LUJV) GPC, making it a candidate drug for treating LUJV infections [176]. Additionally, it was mentioned above that trametinib, as a MEK inhibitor, is also expected to be used for bunyavirus infections. Therefore, trametinib holds significant research value for the treatment of PUUV and SNV. Additionally, the proprotein-processing protease furin inhibitor, decanoyl-Arg-Val-Lys-Arg-chloromethylketone (dec-RVKR-cmk), exhibits antiviral activity [196] and is a potential drug for treating SARS-CoV-2 and HBV [197]. Furthermore, research indicates that decanoyl-RRLL-chloromethylketone (dec-RRLL-cmk), the inhibitors of the cellular proprotein convertase site 1 protease (S1P), targeting GPC, can specifically block the transmission and generation of lymphocytic choriomeningitis virus (LCMV) [198]. Developing drugs as inhibitors of GPC’s processing protease holds promise for treating PUUV or SNV infections.

#### 4.2.4. Gn

Gn and Gc play a role in IFN suppression mechanisms in HTNV and PUUV, which are potential targets for antiviral therapy. However, there is currently a lack of reported treatment strategies specifically targeting the viral Gn or Gc. Research suggests that 2-deoxy-D-glucose (2-DG), the glycolysis inhibitor, inhibits LCMV propagation by targeting glycoprotein N-glycosylation [177]. Additionally, the Drug Controller General of India gave emergency approval for 2-DG in COVID-19 patients, and 2-DG exhibits antiviral activity against HPV18, rhinoviruses (RV), HBV, HSV, etc [178]. Therefore, 2-DG is a promising drug for treating HTNV or PUUV infections, requiring further exploration and validation.

## 5. Conclusions

Some species of the order Bunyavirales exert a significant impact on public health. IFN response suppression is a crucial mechanism for immune evasion following the invasion of the host by bunyaviruses which is a critical determining factor in the pathogenicity. Within this viral order, the targets involved in IFN inhibition exhibit similarities while also presenting certain differences. A large number of potential therapeutic approaches against the IFN response suppression of bunyavirus are presented in this article, which provide references for both the following basic research and practical applications. The research on drugs or compounds targeting the IFN response pathway may potentially offer novel choices and perspectives for antiviral treatments. According to their drug development, antiviral research, and clinical application, we conclude that trametinib, tamoxifen, nitazoxanide, acitretin, salidroside, curcumin, genistein, MSCs, radiotherapy, and DNA damage agents have great clinical application prospects in the treatment of bunyavirus infection.

## Figures and Tables

**Table 1:** The current research status of the suppressing interferon response mechanisms in Bunyavirales.

Viral Name	Main Targets/ Pathways	Mechanisms for the Suppression of IFN Response	Relevant Amino Acid Sites	References
DBV	TRIM25	NSs → IBs → sequester TRIM25 → RIG-I activity ↓	\	[49,50]
	TBK1, IKKε	NSs → IBs → sequester TBK1 and IKKε → TBK1/IKKε-IRF3/IRF7 signaling ↓	Amino acids serine 21 and leucine 23 in NSs.	[51,52]
	IRF7	NSs → IBs → nuclear translocation of IRF7 ↓	\	[54]
	STAT2	NSs → IBs → sequester STAT2, but not STAT1 → phosphorylation and nuclear translocation of STAT2 ↓	\	[53]
	STAT1, STAT2	NSs → IBs → sequester STAT2 and STAT1 → phosphorylation of STAT2 ↓, nuclear translocations of STAT2 ↓ and STAT1 ↓	\	[55]
	LSm14A	NSs → LSm14A → RIG-I activity ↓	The second arginine in the LRRD at the N-terminus of NSs.	[56]
GTV	TBK1, STAT2	NSs → IBs, FSs → sequester TBK1 and STAT2	\	[57]
HRTV	TBK1	NSs → TBK1 → IRF3 activity ↓	Amino acids serine 21 and leucine 23 in NSs.	[52,59]
	STAT1	Without the help of viral protein → STAT1 ↓	\	[58]
	STAT2	NSs → STAT2 → nuclear translocation of STAT2 ↓	\	[58]
NSDV	STAT1, STAT2	L protein → phosphorylation of STAT1 ↓ and STAT2 ↓	\	[60]
RVFV	Transcription factor IIH complex, PKR	NSs → SAP30, YY1 → transcription factor IIH complex ↓, PKR ↓	\	[63,64,65]
	RIOK3	Viral infection → RIOK3 ↓	\	[69]
SFSV	IRF3	NSs → shield the DNA-binding domain of IRF3 → TBK1-IRF3 ↓	\	[71]
	JAK1	NSs → JAK1 → phosphorylation and nuclear translocation of STAT1 ↓ in type I IFN response	\	[72]
	PKR	NSs → eIF2B activity ↑ → translational inhibition effect of PKR ↓	\	[73]
HTNV	MAVS	Gn → TUFM → degrade MAVS	\	[77]
	NF-κB	NP → sequester NF-κB in the cytoplasm, NF-κB activity ↓	\	[74]
	NF-κB	NP → miR-146a ↑ → NF-κB pathway ↓	\	[75]
	NF-κB	NP, Gn, Gc → NF-κB activity ↓, ISGs expression ↓	\	[76]
PUUV	\	GPC, Gn/Gc → IFN-β promoter activity ↓	\	[78]
	ISRE	GPC → antagonizes ISRE on ISGs	\	[78]
	IRF3	NSs → IRF3 activity ↓, IFN-β promoter activity ↓	\	[80]
TULV	IRF3	NSs → IRF3 activity ↓, IFN-β promoter activity ↓	\	[80]
SNV	JAK-STAT	GPC → JAK-STAT ↓	\	[79]
BUNV	RNAP II	NSs → MED8, p44 → dysregulate RNAP II	\	[82]
LACV	RNAP II	NSs → DNA damage response pathway → degrade IIo-borne RBP1 subunits → RNAP II ↓ NSs and cofactor Elongin C → degrade RNAP II subunit RPB1 → RNAP II ↓	\	[84,85]
Phleboviruses	ISG20	Escape the antiviral effect of ISG20	\	[87]
CCHFV	ISG15	L protein → ISG15-encoded ubiquitin ↓, the conjugation of ubiquitin-like proteins with cellular proteins ↓	\	[60]

Note: \ No research indicated amino acid sites of viral proteins associated with the corresponding IFN suppression mechanism; DBV, Dabie bandavirus; GTV, Guertu virus; HRTV, Heartland virus; NSDV, Nairobi sheep disease virus; BUNV, Bunyamwera orthobunyavirus; LACV, La Crosse encephalitis virus; RVFV, Rift Valley fever virus; SFSV, Sandfly fever Sicilian virus; HTNV, Hantaan virus; PUUV, Puumala virus; TULV, Tula virus; SNV, Sin Nombre virus; CCHFV, Crimean–Congo hemorrhagic fever virus; RNAP II, RNA polymerase II; ISRE, IFN-stimulated response element; NSs, the non-structural proteins; PKR, protein kinase R; RIOK3, RIO kinase 3; MED8, mediator complex subunit 8; RBP1, retinol-binding protein type 1; TUFM, mitochondrial Tu translation elongation factor; LC3B, noncanonical light chain 3B; Gn, glycoprotein n; NP, nucleocapsid protein; Gc, glycoprotein C; GPC, glycoprotein precursor; ISGs, IFN-stimulated genes; IBs, inclusion bodies; FSs, filamentous structures; SAP30, Sin3A-associated protein 30; YY1, Yin Yang 1, an oncogenic transcription factor; and LRRD, leucine-rich repeat domain.

**Table 2:** Antiviral research and potential strategies based on key targets of IFN response pathways and viral proteins.

Key Targets	Potential Strategies against Bunyavirus	Research and Applications of Antiviral Therapy	Reference
RLR			
RIG-I and MDA5	PolyI: C; BO-112	Mengo virus, RSV, SARS-CoV-2	[89,90,91,92]
	MSCs	HIV, HBV, SARS-CoV-2	[93,94,95,96,97,98]
RIG-I	NTZ	Influenza virus, Viral gastroenteritis, RV, EBOV	[99,100,101,102]
	Acitretin	HIV, BKPyV, HPV	[103,104,105]
	Salidroside	DENV, CVB3, IAV	[106,107,108,109]
	RNA agonists of RIG-I: 3p10LG9, 5′-pppRNA, 5′-ppp siRNA, 3p-siRNA-MDR1, and SLR14	IAV, DENV, HSV-1, SARS-CoV-2	[110,111,112,113,114,115]
	IR; DNA-damaging agents: doxorubicin, etoposide, teniposide, and oxaliplatin	ZIKV, Influenza virus, Poliovirus, SARS-CoV-2, DENV	[116,117,118,119,120,121,122]
MDA5	DNA demethylation agents: DNMTis such as AZA and DAC	\	[123,124,125]
TBK1	Lidocaine	IAV, SARS-CoV-2	[126]
IRF3	Isoflavone and isoflavone-like compounds: KIN 101, genistein	HCV, Influenza virus, AdV, HSV, HIV, RV	[127,128]
	Hydroxyquinoline family compounds: KIN1400, KIN1408, KIN1409	WNV, DENV, HCV, IAV, RSV, NiV, LASV, EBOV	[129]
Others	P97 inhibitors: clotrimazole	Poliovirus, HSV, CMV, Influenza virus	[130,131]
	ERRα inverse agonist: XCT790; ERRα inhibitors: compound A, kaempferol, compound B, LingH2-10, naringenin, SR16388, five anticancer drugs and nine pesticides that inhibit ERRα activity	CMV, VSV, NDV, HSV, HBV	[132,133,134,135,136,137,138,139,140,141]
	7-DHC; DHCR7 inhibitors: tamoxifen, AY9944, haloperidol, aripiprazole, cariprazine, trazodone, BM15.766	ZIKV, VSV, HCV, HSV, SARS-CoV-2	[142,143,144,145,146,147,148,149,150]
	SOCS antagonist: pJAK2 (1001–1013)	HSV-1, IAV, VV, EMCV, DENV, ZIKV, WNV, EBOV	[151,152,153,154]
	MEKi: trametinib	IAV, RV2, RSVA2	[155,156,157]
NSs	RNAi	RVFV, IAV, PRRSV, WNV, SARS-CoV-2	[158,159,160,161,162]
NP	doxycycline and minocycline	CCHFV	[163]
	curcumin	IAV, EBOV, HIV, DENV, HSV-2, etc.	[164,165,166]
	naproxen	IAV, SARS-CoV-2	[167,168]
	compound RK424	IAV	[169]
	RNAi	hMPV, RABV, BoDV-1, IAV	[170,171,172,173]
	NP-specific mAbs	IAV	[174]
	antisense oligonucleotides	AIV H5N1	[175]
GPC	trametinib	LUJV	[176]
Gn	2-DG	LCMV, SARS-CoV-2, HPV18, RV, HBV, HSV, etc.	[177,178]

Note: \, No research; MSCs, mesenchymal stromal cells; NTZ, nitazoxanide; IR, ionizing radiation; DNMTis, DNA methyltransferase inhibitors; AZA, 5-azacytidine; DAC, 5-aza-2′-deoxycytidine; compound A, N-[(2Z)-3-(4,5-dihydro-1,3-thiazol-2-yl)-1,3-thiazolidin-2-ylidene]-5H dibenzo[a,-d] [7]annulen-5-amine; compound B, cyclohexylmethyl-(1-p-tolyl-1H-indol-3-ylmethyl)-amine; 7-DHC, 7-dehydrocholesterol; DHCR7, 7-dehydrocholesterol reductase; MEKi, MEK inhibitor; RNAi, RNA interference; 2-DG, 2-deoxy-D-glucose; RSV, respiratory syncytial virus; HBV, hepatitis B virus; RV, rotavirus; EBOV, Ebola virus; BKPyV, BK polyomavirus; HPV, human papillomavirus; CVB3, coxsackievirus B3; IAV, influenza A virus; DENV, dengue virus; HSV-1, herpes simplex virus 1; ZIKV, zika virus; HCV, hepatitis C virus; HSV, herpes simplex virus; AdV, adenovirus; WNV, West Nile virus; NiV, Nipah virus; LASV, Lassa virus; PRRSV, porcine reproductive and respiratory syndrome virus; VSV, vesicular stomatitis virus; RV2, respiratory viruses rhinovirus; RSVA2, respiratory syncytial virus; CMV, cytomegalovirus; NDV, newcastle disease virus; VV, vaccinia virus; EMCV, encephalomyocarditis virus, CCHFV, Crimean–Congo hemorrhagic fever virus; hMPV, human metapneumovirus; RABV, rabies virus; BoDV-1, Borna disease virus; AIV, avian influenza virus; LUJV, Lujo virus; LCMV, lymphocytic choriomeningitis virus; and RV, rhinoviruses.
